# Study protocol: Effects of active versus passive recharge burst spinal cord stimulation on pain experience in persistent spinal pain syndrome type 2: a multicentre randomized trial (BURST-RAP study)

**DOI:** 10.1186/s13063-022-06637-7

**Published:** 2022-09-05

**Authors:** Martijn R. Mons, Caro Edelbroek, Xander Zuidema, Katja Bürger, Lars Elzinga, Jessica de Vries, Sander van Kuijk, Elbert A. Joosten, Jan-Willem Kallewaard

**Affiliations:** 1 Department of Anesthesiology and Pain Management, University Pain Clinic Maastricht (UPCM) Maastricht University Medical Center (MUMC+), Maastricht, the Netherlands; 2 Department of Translational Neuroscience, School for Mental Health and Neuroscience (MHeNS), University of Maastricht, Maastricht, the Netherlands; 3 Department of Anesthesiology, Rijnstate Hospital Arnhem, Arnhem, the Netherlands; 4 Department of Anesthesiology, Diakonessen Hospital Utrecht, Utrecht, the Netherlands; 5 Department of Anesthesiology, Alrijne Hospital Leiderdorp, Leiderdorp, the Netherlands; 6 Department of Anesthesiology, Bravis Hospital Roosendaal, Roosendaal, the Netherlands; 7 Department of Anesthesiology, Elizabeth TweeSteden Hospital Tilburg, Tilburg, the Netherlands; 8 Department of Clinical Epidemiology and Medical Technology Assessment, Maastricht UMC+, Maastricht, the Netherlands; 9 Departement of Anesthesiology, Amsterdam Universitair Medisch Centrum, Amsterdam, the Netherlands

**Keywords:** Neuromodulation, Burst SCS, Spinal cord stimulation, Persistent spinal pain syndrome type 2, Pain relief, Motivational-emotional aspects of pain

## Abstract

**Background:**

Spinal cord stimulation (SCS) has shown to be an effective treatment for patients with persistent spinal pain syndrome type 2 (PSPS Type 2). The method used to deliver electrical charge in SCS is important. One such method is burst stimulation. Within burst stimulation, a recharge pattern is used to prevent buildup of charge in stimulated tissues. Two variations of burst waveforms are currently in use: one that employs active recharge and one that uses passive recharge. It has been suggested that differences exist between active and passive recharge paradigms related to both efficacy of pain relief and their underlying mechanism of action. Active recharge has been shown to activate both the medial spinal pathway, engaging cortical sensorimotor areas involved in location and intensity of pain, and lateral pathway, reaching brain areas involved with cognitive-emotional aspects of pain. Passive recharge has been suggested to act via modulation of thalamic neurons, which fire in a similar electrical pattern, and thereby modulate activity in various cortical areas including those related to motivational and emotional aspects of pain. The objective of this randomized clinical trial is to assess and compare the effect of active versus passive recharge Burst SCS on a wide spectrum of pain in PSPS Type 2 patients.

**Methods:**

This multicentre randomized clinical trial will take place in 6 Dutch hospitals. PSPS Type 2 patients (*n=94*) will be randomized into a group receiving either active or passive recharge burst. Following a successful trial period, patients are permanently implanted. Patients complete the Pain Catastrophizing Scale (PCS) (primary outcome at 6 months), Numeric Pain Rating Scale (NRS), Patient Vigilance and Awareness Questionnaire (PVAQ), Hospital Anxiety and Depression Scale (HADS), Quality of Life (EQ-5D), Oswestery Disability Index (ODI), Patient Global Impression of Change (PGIC) and painDETECT questionnaires (secondary outcomes) at baseline, after trial, 1, 3, 6 and 12 months following implantation.

**Discussion:**

The BURST-RAP trial protocol will shed light on possible clinical differences and effectivity of pain relief, including emotional-motivational aspects between active and passive burst SCS in PSPS Type 2 patients.

**Trial registration:**

ClinicalTrials.gov registration: NCT05421273. Registered on 16 June 2022. Netherlands Trial Register NL9194. Registered on 23 January 2021.

## Administrative information

**Table:** 

Data category	Information
Primary registry and trial identifying number	Clinicaltrials.gov: NCT05421273, registered Netherlands Trial Register NL9194, registered
Date of registration in primary registry	23 January 2021
Secondary identifying numbers	n/a
Source(s) of monetary or material support	Abbott Laboratories
Primary sponsor	Abbott Laboratories
Secondary sponsor(s)	N/A
Contact for public queries	MRM m.mons@maastrichtunversity.nl, JWK jkallewaard@rijnstate.nl
Contact for scientific queries	MRM m.mons@maastrichtunversity.nl, JWK jkallewaard@rijnstate.nl
Public title	N/A
Scientific title	Effects of Active Versus Passive Recharge Burst Spinal Cord Stimulation on Pain Experience in Persistent Spinal Pain Syndrome Type 2: A Multicenter Randomized Trial (BURST-RAP study)
Countries of recruitment	The Netherlands
Health condition(s) or problem(s) studied	Persistent Spinal Pain Syndrome Type 2
Intervention(s)	Passive recharge burst (Burst DR^TM^) spinal cord stimulation
	Active recharge burst spinal cord stimulation
Key inclusion and exclusion criteria	Ages eligible for study: ≥18 years Sexes eligible for study: both
	Inclusion criteria Subjects between 18 and 65 years of age At least moderate level of catastrophizing as measured with the Pain catastrophizing scale (PCS) of at least 20. History consistent with PSPS Type 2 of at least 6 months
	Exclusion criteria Subject is unable to operate the device Severe spinal column degeneration likely to cause technical problems with neuromodulation, to be assessed by the treating physician
Study type	Investigator initiated multicenter randomized clinical trial
	Allocation: Unblinded randomization
	Primary purpose: Pain Experience
Date of first enrolment	February 2022
Target sample size	94
Recruitment status	Recruiting
Primary outcome(s)	Change on the PCS scale
Key secondary outcomes	Change in low back and leg pain intenstity, PVAQ, HADS, EQ-5D, ODI, PGIC, analgesia intake and PainDETECT rates

## Background

Spinal cord stimulation (SCS) is increasingly being used for the treatment of patients with intractable chronic pain that are resistant to conventional treatment options [1]. SCS has been shown to reduce pain in patients with intractable chronic pain, improve quality of life and reduce use of analgesics, while maintaining a low rate of adverse effects [2]. The most common indication for spinal cord stimulation is persistent spinal pain syndrome type 2 (PSPS Type 2), previously referred to as failed back surgery syndrome (FBSS) [3–9]. PSPS Type 2 is marked by radicular neuropathic or neuroplastic leg or arm pain, sometimes combined with low back pain (LBP), resulting in a significant decline in their quality of life, psychological outlook and work productivity [3, 10–16].

Patients receiving conventional SCS, characterized by electrical pulses delivered in the 40–60Hz stimulation frequency range, experience paresthesia or a tingling sensations in the painful area. Burst SCS is a new stimulation paradigm that has been suggested to be even more effective compared to conventional SCS and is now approved worldwide [17]. Furthermore, burst SCS eliminates or greatly reduces the incidence of paresthesia [18], hinting at a different underlying mechanism of action as compared to conventional SCS [19]. Burst SCS consist of delivering groups of electrical pulses (also referred to as “burst trains”) which are repeated at a burst rate; within each burst train, several pulses are issued at an intra-burst rate [20]. Following each pulse a recharge pattern is required to prevent the buildup of charge in the stimulated tissue. Individual pulses are characterized by a pulse amplitude and pulse width.

Currently, two variations of burst-SCS are used in treatment of patients with intractable chronic pain. These two variations differ with respect to the recharge pattern employed in the burst train: on the one hand a burst paradigm employing passive recharge (referred to as BurstDR, developed by Abbott Laboratories [20, 21]) and on the other hand using an active recharge pattern (used by Boston Scientific [22–24]).

It has been suggested that differences exist between active and passive recharge Burst SCS paradigms related to both efficacy of pain relief and their underlying mechanism of action [22, 24–30]. Active recharge Burst SCS has been shown, at least in an animal model of chronic neuropathic pain, to activate both the medial spinothalamic pathway, engaging cortical sensorimotor areas involved in location and intensity of pain, as well as the lateral spinothalamic pathway, reaching brain areas involved with cognitive-emotional aspects of pain [26, 31]. Passive recharge has been suggested to act via modulation of thalamic neurons, which fire in a similar electrical pattern, and thereby modulate activity in various cortical areas including those related to motivational and emotional aspects of pain [32]. This is reflected by clinical research which suggest the involvement of such motivational-emotional structures [33–35]. There have been several clinical studies which have described the effect of passive and active burst stimulation in chronic pain in isolation; however, none has directly compared both waveforms on effectivity of pain relief and motivational-emotional aspects of pain in the same patient population [17, 24, 33, 36, 37].

Therefore, the objective of this randomized clinical trial is to assess and compare effect of passive recharge burst SCS with active recharge burst SCS on pain relief and motivational-emotional facets of pain in PSPS Type 2. Due to the lack of direct evidence regarding clinical variations between the two waveforms, we hypothesize that there is no difference in effectivity on pain catastrophizing between Burst SCS with passive recharge as compared to Burst SCS with active recharge in PSPS Type 2 patients, 6 months after the initial implantation.

As the different Burst paradigms may eventually differ in modulation of brain areas involved with emotional-motivational aspects of pain, the primary outcome of this study is change on the Pain Catastrophizing Scale (PCS) at 6 months. The PCS is a questionnaire which aims to chart emotional aspects of pain [4, 38].

The secondary objectives of this study are to clinically compare passive recharge burst SCS with active recharge burst SCS on both functional and emotional aspects of pain as well as consumption of electrical power, related to battery life in order to gain further insight in clinical performance.

## Materials and methods

This multicentre randomized clinical trial will be undertaken in various Dutch Hospitals including the Rijnstate Hospital (Arnhem), Bravis Hospital (Roosendaal), Elizabeth TweeSteden Hospital (Tilburg), Diakonessen Hospital (Utrecht), Alrijne Hospital (Leiden) and the Amsterdam University Medical Hospitals (A-UMC). This protocol has been designed following Standard Protocol Items: Recommendations for Interventional Trials (SPIRIT) reporting guidelines [39].

### Patient population

Subjects are selected based on inclusion and exclusion criteria as displayed below. In order to detect changes in emotional state, patients must show at least a moderate level of pain catastrophizing (PCS ≥ 20) as based on research including catastrophizing PSPS Type 2 patients [4].

### Inclusion criteria

- Subjects between 18 and 70 years of age.
- At least moderate level of catastrophizing as measured with the Pain Catastrophizing Scale (PCS) of at least 20 at first visit of the pain clinic.
- Chronic pain diagnosed as PSPS Type 2 for at least 6 months.
- Neurologic exam without marked motor deficit.
- LBP and/or leg pain intensity should be 5 or higher measured with the 11-box NRS 0-10.
- Meets all the inclusion criteria for the implantation of a neurostimulation system as typically utilized in the study centre. Depression is not an exclusion criteria.
- Subject has been screened by a multi-disciplinary panel including a psychologist and deemed suitable for implantation.
- Subject is able and willing to comply with the follow-up schedule and protocol.
- Subject is able to provide written informed consent.

### Exclusion criteria

- Female subject of childbearing potential is pregnant/nursing or plans to become pregnant during the course of the study.
- Escalating or changing pain condition within the past month as evidenced by investigator examination.
- BMI ≥ 35.
- Subject has had injection therapy or radiofrequency treatment for their LBP or leg pain within the past 3 months.
- Subject currently has an active implantable device including ICD, pacemaker, spinal cord stimulator, or intrathecal drug pump.
- Subject is unable to operate the device.
- Severe spinal column degeneration likely to cause technical problems with neuromodulation, to be assessed by the treating physician.
- Previous neurostimulation therapy.

### Recruitment and consent

Subjects will be recruited from the site’s existing patient population and through new patient contacts through standard clinical practice. Patients will be asked to participate by their physician and presented with the patient information and informed consent form. Patients are given at least a week to consider their decision. At all times, subject are able to contact the research team or an independent specialist with questions. Patients are able to withdraw from the study at any time and without giving a reason to do so.

### Study procedure

 Following inclusion and consent, patients are randomized into two groups: passive burst SCS or active burst SCS. Randomization is performed by local study staff using Castor study management software. Patients are stratified per group and per center for PCS rate (two groups of 20-35 and 35-52), gender and pain location.

Procedures in this study do not deviate from standard neuromodulation care for PSPS Type 2 patients. First, a trial period occurs where patients are implanted with either two Abbott Octrodes or two Boston Scientific Infinion leads in the dorsalepidural space; during the procedure paresthesia testing is performed to achieve maximum overlap with the pain area of the patient. During this trial period, patients are stimulated by an external pulse generator. Stimulation settings are set by clinical programmers provided by Abbott and Boston Scientific, who individually assess optimal burst stimulation parameters for every individual participating patient. Due to the subsequent nature of the study, patients cannot be blinded. Following a test phase of 2 weeks and a minimal pain reduction of 50%, patients are implanted with an internal IPG (Proclaim 5/XR (Abbott Laboratories) or Waverwriter Alpha (Boston Scientific)). When pain reduction is less than 50%, the trial is expanded by 1 week, and other (non-burst) waveforms possible with the implanted system can be tried, with the aim to induce pain relief. These patients, with at least 50% pain relief, will remain in the study and complete long-term follow up questionnaires.

Data regarding pain state and mental attitude towards pain are collected at baseline, after trial, 1 month, 3 months, 6 months and 12 months following implantation. Patients will electronically complete the Pain Catastrophizing Scale (PCS) [38], Numeric Pain Rating Scale (NRS), Patient Vigilance and Awareness Questionnaire (PVAQ) [40, 41], Hospital Anxiety and Depression Scale (HADS) [42, 43], Quality of Life (EQ-5D) [44–46], Oswestery Disability Index (ODI) [47], Patient Global Impression of Change (PGIC) [48] and painDETECT [49, 50] questionnaires using the CASTOR data collection system. Additionally, use of pain medication alongside neuromodulation is recorded at every follow-up contact, as well as the mean charge per second (CpS) and mean charge per hour (CpH) as used by the stimulation apparatus in order to gain insight into electrical consumption.

**Fig. 1:**
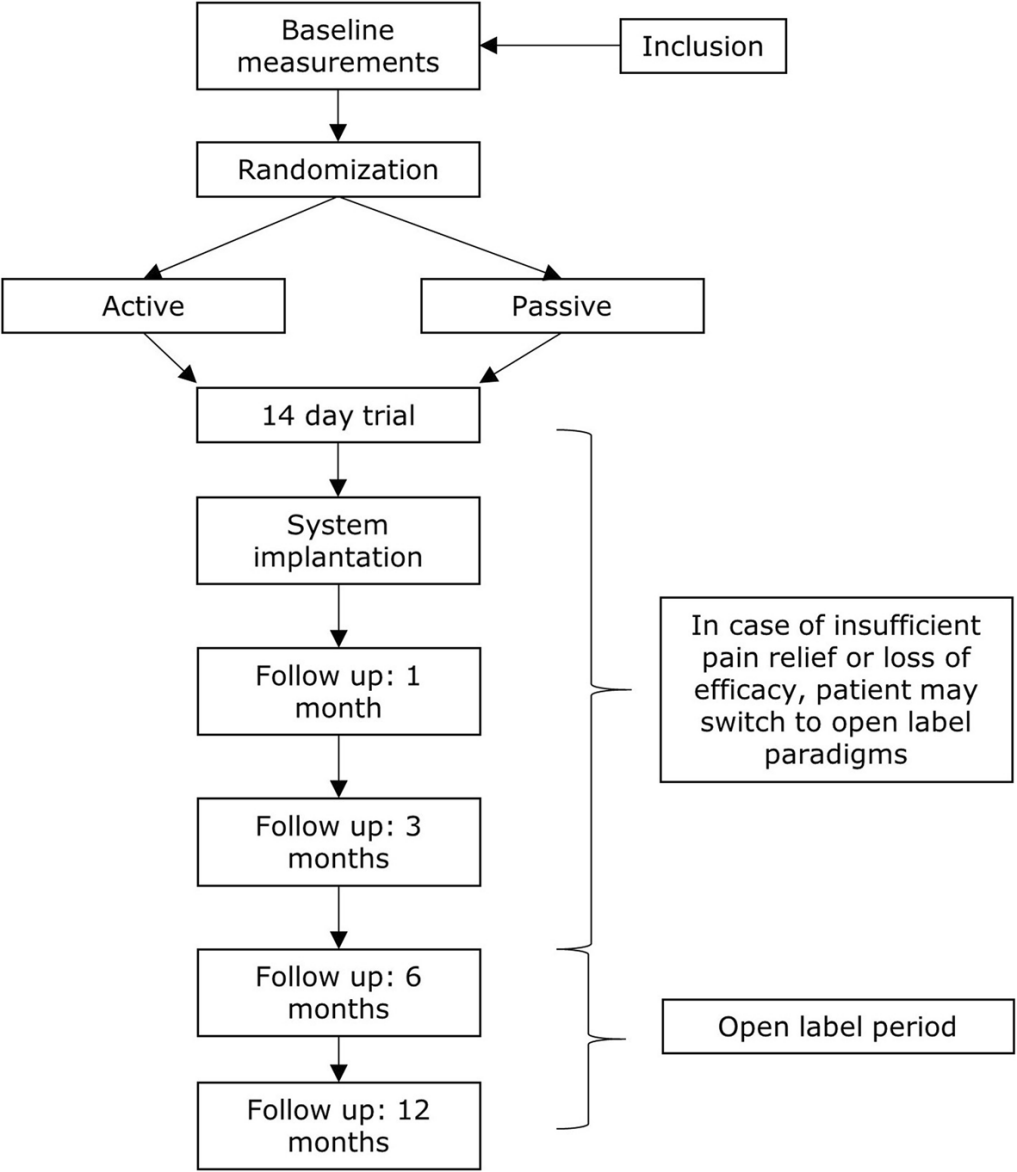
Study flow chart

Six months post implantation, the trial enters an open-label phase, running until 12 months. During this open-label phase, patients can switch to other non-burst waveforms permitted by the SCS system implanted with the aim to further increase pain relief. If, during any of the trial phases including the initial trial, patients encounter decreased efficacy or insufficient pain relief, they may enter the open label phase prematurely. A flow chart outlining study procedures is displayed in Fig. 1 and in Table 1.

**Table 1:** SPIRIT figure of study structure

	Study period					
Timepoint	Enrolment	Implantation	Trial	3 months	6 months	12 months
Enrolment:						
Eligibility screening	X					
Informed consent	X					
Randomization	X					
Intervention:						
Active burst neuromodulation		X	X	X	X	X
Passive burst neuromodulation		X	X	X	X	X
Open-label phase		In case of LOE	In case of LOE	In case of LOE	In case of LOE	X
Assessments:						
PCS	X		X	X	X	X
NRS	X		X	X	X	X
ODI	X		X	X	X	X
PainDETECT	X		X	X	X	X
PVAQ	X		X	X	X	X
HADS	X		X	X	X	X
PGIC	X		X	X	X	X
CPH/CPM				X	X	X

*LOE* loss of effect

Recording of adverse events (AE) and serious adverse events (SAE) is done through the CASTOR data management system.

### Data management

Patients are given an ID number upon entering the study. The list of ID numbers and corresponding patient details is accessible only to specific members of the research staff. The anonymized ID numbers are logged in the password-protected CASTOR data management system, where study records are kept.

### Sample size calculation

This study follows a non-inferiority setup based on the primary outcome measure, the PCS. The non-inferiority limit of 7 points on the PCS scale (13.46%) was defined based on an estimation of the clinically meaningful score of PCS scores established on experience with this test at the main research centre. Previous literature investigating the clinically meaningful difference of the PCS scale estimates that this lies at around 44% [51]. However, as this study was performed in patients with subacute pain after whiplash injury, the decision was made to lower the non-inferiority limit for patients with PSPS Type 2, a chronic debilitating condition with neuropathic components.

In order to calculate group size, an estimation of the expected standard deviation (SD) was based on previous literature for FBSS patients with neuromodulation to be 12.04 points on the PCS scale [4]. In combination with a non-inferiority limit of 7 points, 80% power and 5% significance level, this results in a group size of 37 patients per group or 74 patients total. Compensating for a drop-out rate of 20% (including failed initial trials), we arrive at 92.5 patients. However, this is rounded to 94 patients in order to ensure even group size of 47 patients per group.

### Statistical analysis

All main analyses will be performed according to the intention-to-treat principle. Hence, all patients who do not show sufficient pain reduction in the first phase will still be considered for the analyses. In addition, an exploratory per protocol analysis will be performed on those that do show sufficient benefit in the first phase.

### Primary study parameters

Between-group differences in mean PCS scores at 6 months will be computed including the one-sided 95% confidence interval. The upper bound of the difference will be compared to the non-inferiority margin of 7 points. If the margin lies in the 95% confidence interval, the null-hypothesis that active recharge is not inferior to passive recharge cannot be rejected.

### Secondary study parameters

changes in LBP intensity, PVAQ, HADS, EQ-5D, ODI, PGIC and painDETECT rates will be compared between groups using the independent-samples *t*-test. Group differences in subject satisfaction with treatment will be compared using the Mann-Whitney *U* test. The percentage of subjects who achieve a reduction inLBP intensity of ≥50% and 30% compared to baseline and the percentage of patients experiencing AE’s or SAE’s will be compared between groups using Pearson’s chi-squared test. In case of expected cell counts of 5 or lower, Fisher’s exact test will be used. Analgesia intake changes between the 2 groups and incidence of AE or SAEs will be assessed using descriptive statistics only.

### Data monitoring committee

As this protocol adheres to standard treatment for PSPS Type 2 patients undergoing neuromodulation, no extra risk to patients is expected. As such, no data monitoring committee has been formed for this study.

### Monitoring

This study will be monitored through the Rijnstate’s monitoring programme, independent from the investigational team and according to the Dutch Medical Research Involving Human Subjects Act (WMO). This includes monitoring of informed consent forms and adherence to inclusion and exclusion criteria (per centre: first three inclusions, then 10% randomly selected), source data verification (10% at random), reporting of SAEs (10% at random) and monitoring of study material and study procedures at every site, both digitally and on-site.

## Conclusions

The BURST-RAP protocol is designed to study the effect of active recharge Burst SCS as compared to passive recharge Burst SCS on pain experience in PSPS Type 2 patients. The results from this study may provide clinicians with more information on effectivity of passive and active recharge Burst SCS on pain catastrophizing, pain relief and various other aspects of pain. Furthermore, as both device performance and patient preference are monitored during this study, this will ultimately contribute to a more optimal pain management for the PSPS Type 2 patient.

### Trial status

This manuscript is based on protocol version V7 21.04.2022. Any following substantial amendments will be notified to the Commissie Mensgebonden Onderzoek (CMO) and to the competent authority for approval. The first patient was included on February 16, 2022, in the Rijnstate Hospital. Intakes in the other centres will continue in the months following.

## Data Availability

Data generated by this trial will be only available to trial researchers for processing.
